# Understanding the Consumption of Antimicrobial Resistance–Related Content on Social Media: Twitter Analysis

**DOI:** 10.2196/42363

**Published:** 2023-06-12

**Authors:** Hyunuk Kim, Chris R Proctor, Dylan Walker, Ronan R McCarthy

**Affiliations:** 1 Department of Management and Entrepreneurship Martha and Spencer Love School of Business Elon University Elon, NC United States; 2 Division of Biosciences, Department of Life Sciences Centre of Inflammation Research and Translational Medicine, College of Health and Life Sciences Brunel University London Uxbridge United Kingdom; 3 The George L Argyros School of Business and Economics Chapman University Orange, CA United States

**Keywords:** antimicrobial resistance, AMR, social media, Twitter, engagement, antimicrobial, effective, public health, awareness, disease, microbiology, pathogen, development

## Abstract

**Background:**

Antimicrobial resistance (AMR) is one of the most pressing concerns in our society. Today, social media can function as an important channel to disseminate information about AMR. The way in which this information is engaged with depends on a number of factors, including the target audience and the content of the social media post.

**Objective:**

The aim of this study is to better understand how AMR-related content is consumed on the social media platform Twitter and to understand some of the drivers of engagement. This is essential to designing effective public health strategies, raising awareness about antimicrobial stewardship, and enabling academics to effectively promote their research on social media.

**Methods:**

We took advantage of unrestricted access to the metrics associated with the Twitter bot @AntibioticResis, which has over 13,900 followers. This bot posts the latest AMR research in the format of a title and a URL link to the PubMed page for an article. The tweets do not contain other attributes such as author, affiliation, or journal. Therefore, engagement with the tweets is only affected by the words used in the titles. Using negative binomial regression models, we measured the impact of pathogen names in paper titles, academic attention inferred from publication counts, and general attention estimated from Twitter on URL clicks to AMR research papers.

**Results:**

Followers of @AntibioticResis consisted primarily of health care professionals and academic researchers whose interests comprised mainly AMR, infectious diseases, microbiology, and public health. Three World Health Organization (WHO) critical priority pathogens—*Acinetobacter baumannii*, *Pseudomonas aeruginosa*, and Enterobacteriaceae—were positively associated with URL clicks. Papers with shorter titles tended to have more engagements. We also described some key linguistic characteristics that should be considered when a researcher is trying to maximize engagement with their publication.

**Conclusions:**

Our finding suggests that specific pathogens gain more attention on Twitter than others and that the levels of attention do not necessarily correspond to their status on the WHO priority pathogen list. This suggests that more targeted public health strategies may be needed to raise awareness about AMR among specific pathogens. Analysis of follower data suggests that in the busy schedules of health care professionals, social media offers a fast and accessible gateway to staying abreast of the latest developments in this field.

## Introduction

Antimicrobial resistance (AMR) is recognized by the World Health Organization (WHO) as one of the most pressing concerns of our time. Overprescription and incorrect usage of antibiotics have further contributed to the worsening crisis. With deaths attributed to AMR reaching 4.95 million in 2019 [1] and a predicted cumulative global cost of $100 trillion by 2050, it is clear that novel strategies to combat AMR are desperately needed [2,3]. Over the last 2 decades, several campaigns have been run both nationally and internationally to raise awareness about AMR and the threat it poses to our global health care infrastructure. This includes events such as “World Antibiotic Awareness Week,” run each year by the WHO, the “Keep Antibiotics Working” campaign run by Public Health England, and the “Antibiotic Guardian” scheme designed to educate the public about AMR [4,5]. These campaigns have had a significant impact, bringing the threat of AMR into the public domain and raising awareness among all demographics.

Social media has also emerged as a powerful tool for information dissemination and is an excellent platform to facilitate the further proliferation of public awareness campaigns. The social media platform Twitter has been leveraged in a number of unique ways to achieve this [6]. A bustling chorus of world-leading scientists uses Twitter to amplify their research and offer up-to-the-minute opinions on key health care issues [7-9], and virtual journal clubs on Twitter are held across a number of specialties [10-12]. One tool that has emerged on Twitter that has a significant legacy impact is linking auto-posting Twitter bots to research publication sites such as the National Center for Biotechnology Information or bioRxiv. Linking these Twitter bots via RSS feeds has enabled scientists around the world to stay abreast of the latest developments in their respective fields within the comfort of a social media application. There are numerous successful examples of these Twitter bots with large followings within the field of microbiology, such as @pseudo_papers, which posts the latest research on the organism Pseudomonas and has over 1200 followers. Twitter bots such as these have curated a following that cuts across research disciplines, age brackets, and professions, as there are no restrictions on who can follow their feeds. Over the last 5 years, they have become an important dissemination platform for the latest research in a given field. A particularly interesting feature of the majority of these bots is that their posts contain only the title of the research article and a link to the full text. This removes any prestige associated with the publishing journal, impact factor, authors, or institutions. Consequently, analysis of the interaction metrics associated with these tweets gives an unbiased and unfiltered insight into what research is generating the greatest impact on social media.

One of the most popular Twitter bots in the field of microbiology is the @AntibioticResis page, which posts the latest antibiotic resistance–associated research. This Twitter bot was founded in 2015 by some of the authors of this study, and in the intervening years, has curated over 13,900 followers. The engagement metrics associated with this page offer unparalleled insights into what AMR-associated research attracts the most attention on social media and which publications are generating the most discussion. In this study, we take an in-depth look at how people engage with AMR research on social media platforms by exploring the interaction and engagement analytics of the leading Twitter bot disseminating AMR research, the @AntibioticResis bot. We uncover what AMR-related phrases drive content interaction on this platform and investigate the links between priority pathogens and social media interest in these pathogens. The individuals and organizations that interact with this content and amplify it through retweeting are also profiled to gain greater insight into how this information percolates through a social media audience.

## Methods

### Overview

We analyzed 2762 tweets created between December 2018 and September 2021. URL clicks available on Twitter analytics were used as a proxy for user interest. Twitter analytics data for @AntibioticResis were collected from the in-built analytics toolset on the Twitter website. Data fields include the number of impressions (the total number of times a tweet was displayed on timelines), engagements (the total number of interactions with a tweet—likes, retweets, and replies), and the number of retweets, replies, likes, and URL clicks.

### Follower Categorization

To understand the impact of user base characteristics on URL clicks, we examined the most common words that occurred in the user biographies of the @AntibioticResis account followers (retrieved on June 17, 2022). To obtain the common words, we removed hyperlinks, special characters, and stopwords from the biographies written in English (inferred from the Python *langdetect* package; Python Software Foundation). Single characters were not considered common words. After applying the above cleaning steps, we identified dominant roles (health practitioners and professionals, academics) and topics (AMR, infectious diseases, microbiology, and public health) from a word co-occurrence network. We then curated a list of words for each role or topic and counted the number of followers by role and topic (Tables S1 and S2 in Multimedia Appendix 1).

### Retrieving Publication and Tweet Counts to Quantify Attention to Pathogens

Follower categories suggest that there are two different types of attention to tweeted scientific articles: (1) academic attention from the followers classified into health practitioners, professionals, and academics and (2) general attention from the followers who do not provide roles. We acknowledge that some followers without role information may actually be health practitioners, professionals, or academics. We assume that this case is uncommon. We termed this academic attention and general attention, respectively. In particular, we focused on 12 WHO priority pathogens (critical: *Acinetobacter baumannii*, *Pseudomonas aeruginosa*, Enterobacteriaceae; high: *Enterococcus faecium*, *Staphylococcus aureus*, *Helicobacter pylori*, *Campylobacter* spp, *Salmonella*, *Neisseria gonorrhoeae*; medium: *Streptococcus pneumoniae*, *Haemophilus influenzae*, and *Shigella* spp) and 3 organisms of specific interest: *Escherichia coli*, *Klebsiella pneumoniae*, and *Enterobacter* spp.

Many organisms receive greater attention from the academic community than others, and this has the potential to impact engagement levels on Twitter. To quantify this attention for the selected pathogens, we obtained the total number of papers published per year as indexed on PubMed with a set of broad search terms to capture all possible cases. Search terms consisted of the full pathogen name or bacterial name with the abbreviated genus and a truncated wildcard to encompass terms related to resistance (eg, “*Acinetobacter baumannii*” OR “*A. baumannii*”) AND resist* AND Journal Article [Publication Type]). Where a whole genus was named, this was used as the search term along with the truncated wildcard search term (eg, “Enterobacter” AND resist*). Data collection was limited to papers published from 1990 to 2021. Academic attention was calculated from the publication shares for pathogens by year. To clarify the steps of measuring academic attention, we introduced the following mathematical notation: suppose *n_i_*_,2021_ is the number of publications for pathogen *i* in the year 2021. The publication shares for *i* in 2021 *p_i_*_,2021_ equal to 
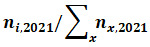
. If a tweet in 2021 contains 2 pathogen names, *i* and *j*, the academic attention on this tweet is *p_i_*_,2021_+*p_j_*_,2021_. The minimum of academic attention is 0 if no pathogen name is listed in a tweet, and the maximum value in our data is 0.496 for the paper titled “Synthesis and characterization of biogenic selenium nanoparticles with antimicrobial properties made by *Staphylococcus aureus*, methicillin-resistant *Staphylococcus aureus*, *Escherichia coli*, and *Pseudomonas aeruginosa*.” The distribution of academic attention values can be seen in Figure S1 in Multimedia Appendix 1.

General attention is likely different from academic attention to the selected pathogens. We inferred the general attention on the Twitter platform from the number of tweets mentioning a pathogen name by using the Twitter Academic application programming interface (API). As tweets have a character limit, it was likely that bacterial names would be shortened, so tweets containing either the full bacterial name or bacterial names with an abbreviated genus were included (eg, “*Pseudomonas aeruginosa*” AND “*P. aeruginosa*”). Unlike PubMed, the Twitter API does not allow wildcard search, so we use a query (“full name” OR “abbreviated name”) (resist OR resistant OR resistance).

### Constructing Phrases From Paper Titles

To identify candidate phrases attracting Twitter accounts to click a URL link, each title was converted into a list of the selected pathogens, organisms, and unigrams from the tweets as follows. First, all uppercase characters were converted to lowercase characters, and then stopwords, single-digit numbers, and special characters except hyphens joining 2 words were removed. Second, the WHO priority pathogens were identified (both full names and abbreviations; eg, *Acinetobacter baumannii* and *A. baumannii*) and considered independent phrases. Third, the remaining words in a title were stemmed from the Porter stemmer [13].

### Negative Binomial Regressions

The URL clicks in our data were overdispersed and heavily right-skewed (Figure S2 in Multimedia Appendix 1). A total of 377 tweets (14%) did not obtain a single URL click, and the maximum number of URL clicks was 66 for the paper titled “The cost of antimicrobial resistance.” The average number and variance of URL clicks were 5.0 and 32.9, respectively. To explain overdispersed URL clicks, we used a negative binomial (NB) regression with identified phrases. The probability of having a count *y* in NB *f*(*Y*=*y*|*λ_i_*,*P*)is written as


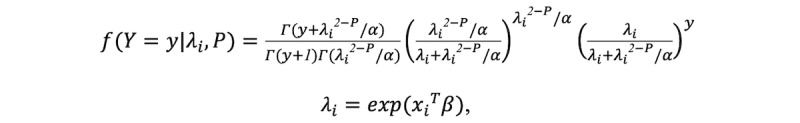


where *λ_i_* is the mean of the distribution, *x_i_* is the covariates of *i*, β is a vector for coefficients, and *P* is the dispersion power parameter, which is set to 1 in this paper. We estimated β for the following covariates to understand the impacts of various factors on URL clicks. The NegativeBinomialP function in the *Statsmodels* Python package was used for our analyses [14].

First, we constructed binary variables indicating whether a title contains a WHO priority pathogen or a common nonpathogen stem appearing in more than 50 tweets (65 common nonpathogen stems exist in total). *x_ik_* is 1 if a pathogen or a stem *k* is mentioned in the tweet *i*, and 0 otherwise. Second, the number of retweets was included as a covariate to consider the size of potential audiences varying by the number of followers of retweeters. Third, the number of phrases in a title was added to the regression models, as long titles may discourage Twitter users from clicking a link. Fourth, we considered academic attention and general attention to the pathogens, as academic attention and general attention may also affect URL clicks in addition to the phrases shown in a title. For each pathogen, we calculated its publication and tweet shares over the selected pathogens for each year in order not to be affected by total publication and tweet counts over time. For each tweet, academic attention and general attention are calculated as the sum of the publication and tweet shares for the pathogens, respectively. Interestingly, academic attention and general attention are highly correlated (the Pearson correlation is 0.93), though they were measured from different sources. Using the covariates above, we built 3 different NB models explaining URL clicks. Model 1 described URL clicks per tweet in the context of the selected phrases, the number of retweets the post received, and the number of individual phrases contained within the title. Each of the following models used all the factors from model 1 with added considerations. Model 2 considered academic attention only, and model 3 considered general attention only.

### The Dispersion of Papers About the WHO Priority Pathogens in a Vector Embedding Space

To generate vector representations of paper titles, a pretrained SPECTER was used [15]. This SPECTER obtained embeddings of scientific articles based on their citations to convert paper titles into vectors. If 2 paper titles appeared close together in the SPECTER embedding space, their topics were likely to be similar. Leveraging these characteristics, for each WHO priority pathogen, the embedding vectors of paper titles containing the pathogen name were obtained.

### Correlation of Article Type and Twitter Engagements

To assess how engagement changes depending on paper type, tweets with ten or more URL clicks (N=380) were manually categorized according to the type of article included in the tweet. Categories used were review articles, original basic research, clinical, and others (including short communications, comments, errata to previously published works, and books). Paper types were expressed as a percentage of the total number of tweets per number of URL clicks.

### Ethical Considerations

No ethics review was sought because the study only explored publicly available data on social media and did not conduct any experiments on human subjects.

## Results

### Who Engages With the @AntibioticResis Account

We aimed to characterize the followers of the @AntibioticResis Twitter account to better understand the users that most frequently engage with this content on social media. Firstly, we assessed the number of new followers gained per month over the time period from July 2016 to April 2022. Interestingly, it was noted that there was an obvious spike in new followers in November each year from 2016 to 2020 (Figure S3 in Multimedia Appendix 1).

To gain a better understanding of the professional backgrounds of the individuals who follow this Twitter bot, a co-occurrence network of frequent words that appear in more than 50 followers’ biographies was created. A node in the network is a keyword, and a link between 2 nodes is created if these nodes appear together in more than 50 followers’ biographies. Links have no weight, so all links have the same width. The length of a link does not represent any quantity of the network but derives instead from the network layout algorithm. The locations of nodes are determined by the ForceAtlas 2 algorithm in Gephi [16]. The ForceAtlas 2 algorithm [17] positions nodes by making them repulse each other based on their connections.

**Figure 1:**
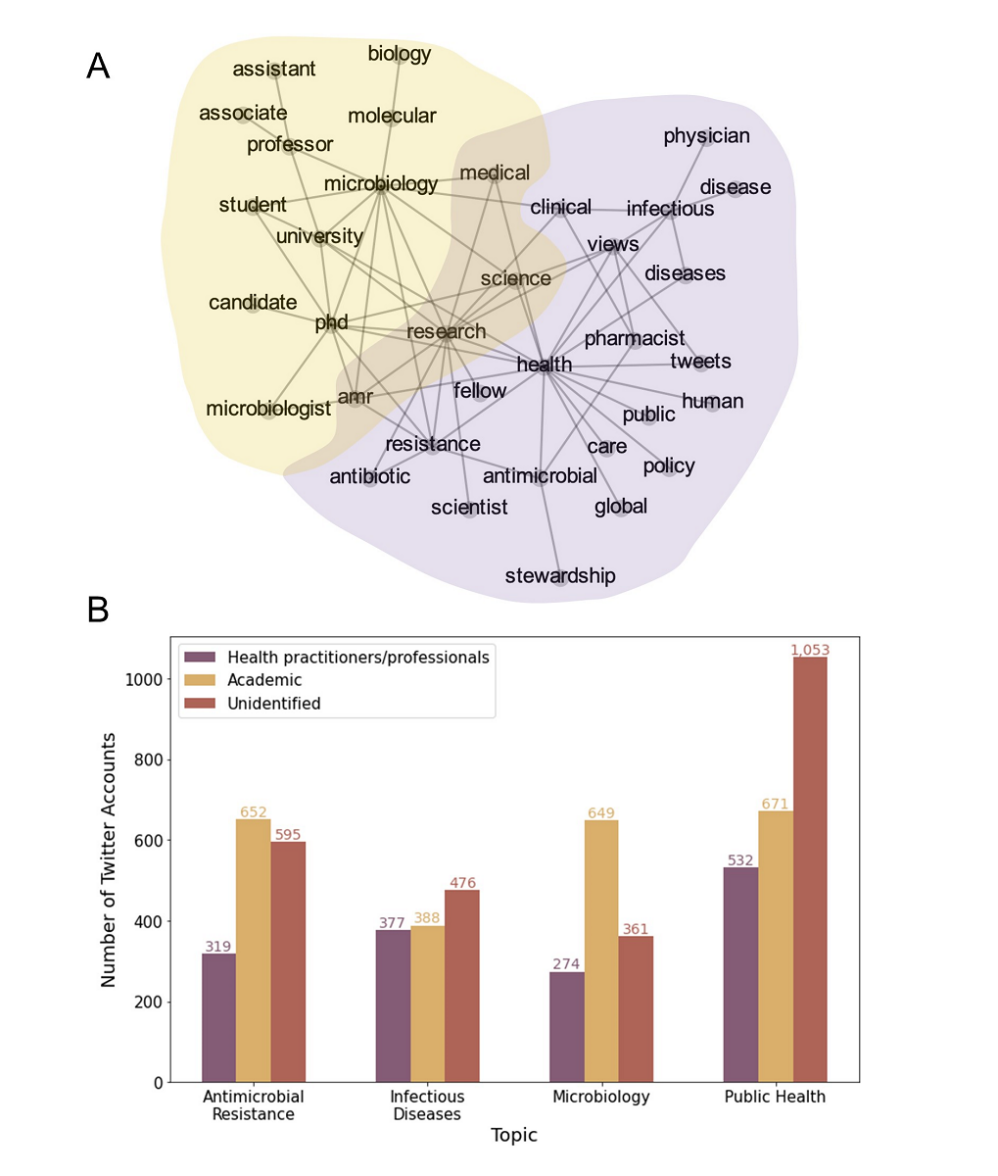
(A) A co-occurrence network of words that appear in the biographies of 50 or more followers. Two words are connected with an unweighted link if they coappear in more than 50 followers’ biographies. Words that are more closely related to clinical or medical practice are highlighted in purple, while words related to basic science are highlighted in yellow. (B) The number of @AntibioticResis followers categorized into clinical, academic, or undefined and further subcategorized by area of interest.

The co-occurrence network reveals that the primary group of individuals following the @AntibioticResis account are likely health practitioners and professionals or academic researchers (Figure 1). These roles tend to be associated with 4 areas: AMR, infectious diseases, microbiology, and public health. The words representing each group (health care professionals and academics) are shown in Table S1 in Multimedia Appendix 1, and the terms related to each topic are listed in Table S2 in Multimedia Appendix 1. A biography can have words across multiple roles and multiple topics. The numbers of followers by role and topic are provided in Figure 1.

### Academic and General Attention to the Selected Pathogens

The WHO has identified several pathogens and bacterial families (divided into critical priority, high priority, and medium priority) for which developing new treatments should be an international prerogative [18]. As priority pathogens, these organisms should receive increased attention as funders and governments recognize the need to support work on them, and the public is widely informed about their negative impacts on health.

**Figure 2:**
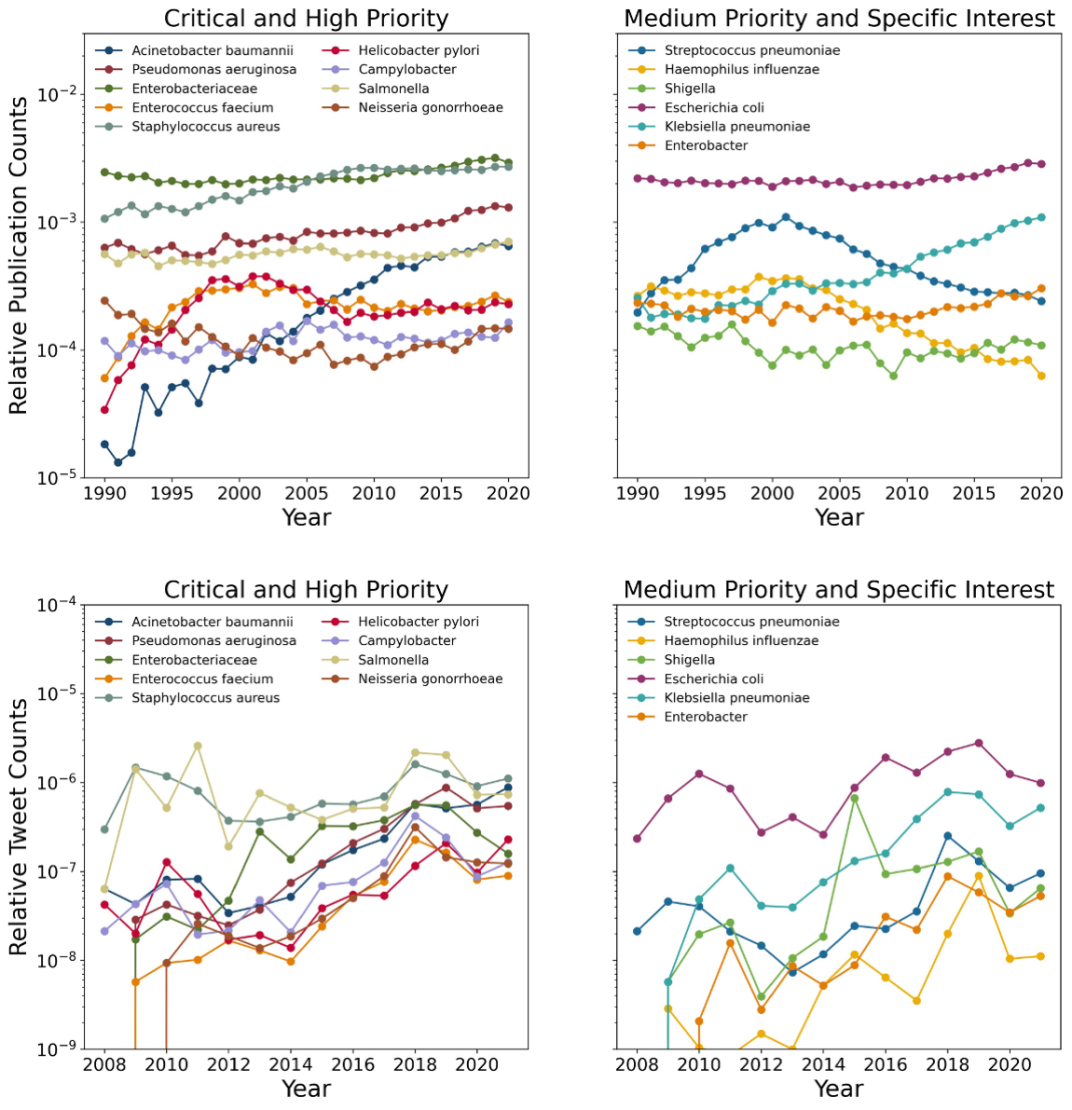
(Top) The relative publication counts for the selected pathogens. For each pathogen, the relative publication counts are calculated as the proportion of the number of journal articles with a pathogen name to the total number of journal articles on PubMed. (Bottom) The relative tweet counts for the selected pathogens. For each pathogen, the relative tweet counts are calculated as the proportion of the number of tweets having a pathogen name to the total number of English tweets from StoryWrangler [17].

To explore academic and general attention for the selected pathogens, we calculated relative publication and tweet counts compared to 2 baselines. The baseline for academic attention is the number of journal articles on PubMed with a query “Journal Article[Publication Type],” and the baseline for general attention is the number of English tweets from StoryWrangler, a comprehensive tool providing daily word usage since 2008 [19] (Figure 2; see Figure S4 in Multimedia Appendix 1 for raw publication and tweet counts for the selected pathogens).

The relative publication counts for *A. baumannii* and *K. pneumoniae* increased, suggesting rapidly growing academic attention to these pathogens. Other critical and high-priority pathogens and organisms of specific interest maintained certain levels of relative publication counts. Two medium-priority pathogens, *S. pneumoniae* and *H. influenzae*, lost academic attention. On the other hand, the relative tweet counts tend to increase for most of the selected pathogens, reflecting an increased general interest. This trend partly reflects increasing awareness about AMR as a result of global and national public health campaigns.

It was noted that some organisms had spikes in general public interest over time. For example, it was shown that the medium-priority organism *Shigella* had a substantial increase in interest from the general public on Twitter in 2015. This spike was shown to coincide with an increase in searches on Google for that organism (Figure S5 in Multimedia Appendix 1). This suggests that increases in the general interest of a particular organism on the internet and discussions surrounding it on social media may be closely related.

### Specific Pathogens Can Positively Influence URL Clicks

The WHO priority pathogen list is recognized as a key scale of the threat posed by each pathogen. We hypothesized that URL clicks would be correlated to the priority level of the pathogens on this list. To explore this, we used NB regressions. Model 1 estimated URL clicks based on the existence of the selected bacterial name, the number of retweets, and the number of phrases in a title. From this model, 3 critical priority pathogens—*A. baumannii*, *P. aeruginosa*, and Enterobacteriaceae—and one high-priority pathogen—*S. aureus*—and 3 pathogens of specific interest—*E. coli*, *K. pneumoniae* (also considered collectively under the term Enterobacteriaceae), and *Enterobacter*—were found to be positively associated with URL clicks (Table 1). This indicates that Twitter users’ interest in AMR partially aligns with the WHO’s priority pathogen list. Furthermore, several pathogens, including *Campylobacter* spp, *N. gonorrhoeae*, and *Salmonella* spp, showed no association with URL clicks. However, the significant coefficients may originate from the time-varying interests of academic researchers and the general audience. For this reason, we include academic attention (model 2) and general attention (model 3) to model 1. In model 2, academic attention is not significant, while adding it increases the rate ratios for *A. baumannii*, *P. aeruginosa*, Enterobacteriaceae, and *K. pneumoniae*, although with less statistical significance. In model 3, general attention is also not significant. The rate ratios and their significance are close to those in model 1 (see Tables S3, S4, and S5 in Multimedia Appendix 1 for the full regression results). These results suggest that the WHO critical priority pathogens and *K. pneumoniae* tend to attract URL clicks more than other selected pathogens, even though we consider both academic and general attention.

**Table 1:** The negative binomial regression results for the selected pathogens with a dependent variable, the number of URL clicks. The rate ratio, which is an exponentiated coefficient, is reported with a 95% CI. “Tweets” refers to the number of tweets containing a pathogen name in the title. The total sample size is 2762 (model 1: stems, number of retweets, and number of phrases in a title; model 2: model 1 with academic attention; and model 3: model 1 with general attention).

Pathogen	Tweets, n	WHO^a^ priority	Rate ratio (95% CI)					
			Model 1	*P* value	Model 2	*P* value	Model 3	*P* value
*Acinetobacter baumannii*	66	Critical	1.39 (1.18-1.64)	<.001	2.03 (1.15-3.59)	.01	1.69 (1.26-2.27)	<.001
*Pseudomonas aeruginosa*	79	Critical	1.33 (1.13-1.56)	<.001	2.83 (0.95-8.38)	.06	1.47 (1.12-1.94)	.006
Enterobacteriaceae	47	Critical	1.83 (1.52-2.21)	<.001	9.51 (0.92-98.37)	.06	2.02 (1.54-2.63)	<.001
*Enterococcus faecium*	10	High	1.11 (0.71-1.72)	.66	1.27 (0.78-2.07)	.33	1.11 (0.63-1.96)	.73
*Staphylococcus aureus*	124	High	1.33 (1.16-1.51)	<.001	6.03 (0.70-52.10)	.10	1.51 (1.05-2.17)	.03
*Helicobacter pylori*	41	High	0.81 (0.60-1.08)	.15	0.91 (0.65-1.29)	.61	0.92 (0.68-1.23)	.56
*Campylobacter*	12	High	0.79 (0.47-1.30)	.35	0.85 (0.51-1.42)	.53	0.69 (0.40-1.21)	.20
*Salmonella*	58	High	1.07 (0.86-1.34)	.55	1.59 (0.87-2.92)	.13	1.25 (0.82-1.91)	.29
*Neisseria gonorrhoeae*	17	High	0.74 (0.49-1.11)	.14	0.79 (0.52-1.21)	.28	0.74 (0.47-1.15)	.18
*Streptococcus pneumoniae*	13	Medium	1.01 (0.62-1.63)	.98	1.16 (0.69-1.95)	.58	0.95 (0.57-1.59)	.84
*Haemophilus influenzae*	3	Medium	0.28 (0.04-1.85)	.19	0.29 (0.04-1.94)	.20	0.44 (0.12-1.59)	.21
*Shigella*	9	Medium	0.94 (0.53-1.68)	.84	1.01 (0.56-1.82)	.97	1.00 (0.54-1.84)	.99
*Escherichia coli*	162	—^b^	1.28 (1.13-1.44)	<.001	6.45 (0.64-64.54)	.11	1.50 (0.90-2.53)	.12
*Klebsiella pneumoniae*	79	—	1.61 (1.40-1.86)	<.001	3.01 (1.23-7.33)	.02	1.70 (1.33-2.18)	<.001
*Enterobacter*	9	—	1.54 (0.96-2.46)	.07	1.79 (1.07-3.00)	.03	1.39 (0.78-2.49)	.26
Number of retweets	—	—	1.24 (1.22-1.26)	<.001	1.24 (1.22-1.26)	<.001	1.28 (1.25-1.31)	<.001
Number of phrases	—	—	0.93 (0.92-0.94)	<.001	0.93 (0.92-0.94)	<.001	0.93 (0.92-0.94)	<.001
Academic attention	—	—	—	—	0.00 (0.00-25.88)	.17	—	—
General attention	—	—	—	—	—	—	0.60 (0.07-4.99)	.63
Total sample size	—	—	2762	—	2762	—	2762	—
Pseudo *R*^2^	—	—	0.07	—	0.07	—	0.07	—

^a^WHO: World Health Organization.

^b^Not available or applicable.

### Nonpathogen Specific Covariates Can Significantly Increase or Decrease URL Clicks

The mention of specific pathogens was expected to affect URL clicks. However, the impact of nonpathogen-specific words and phrases (stems) on URL clicks was unclear. We, therefore, examined common nonpathogen-related stems from the titles of the papers tweeted by the @AntibioticResis account. In model 1, a number of nonpathogen-related stems were found to have either a significant increase or significant decrease in URL clicks associated with their presence in an article title (see Table S3 in Multimedia Appendix 1 for all estimates). Those that significantly (*P*<.05) increased URL clicks were “gram-neg” (rate ratio: 1.47, *P*<.001), “antimicrobi” (1.44, *P*<.001), “urinari” (1.41, *P*=.048), “new” (1.35, *P*=.001), “bacteria” (1.35, *P*<.001), “use” (1.27, *P*=.005), “emerg” (1.25, *P*=.01), “treatment” (1.24, *P*<.001), “antibiot” (1.24, *P*<.001), “detect” (1.21, *P*=.02), “molecular” (1.19, *P*=.04), “multidrug-resist” (1.18, *P*=.02), and “resist” (1.13, *P*=.002). Nonpathogen stems that significantly (*P*<.05) reduced URL clicks were “activ” (0.81, *P*=.007), “agent” (0.73, *P*=.02), and “viru” (0.73, *P*=.045). The number of retweets was positively associated with URL clicks (1.24, *P*<.001), implying, as expected, that retweets enhance the visibility of tweets. On the other hand, the number of phrases in a paper was found to be negatively associated with URL clicks (0.93, *P*<.001). This suggests that papers with longer titles are less likely to encourage Twitter users to click a link.

### Are Organisms With Increased URL Clicks Published Under a Diverse Range of Topics?

**Figure 3:**
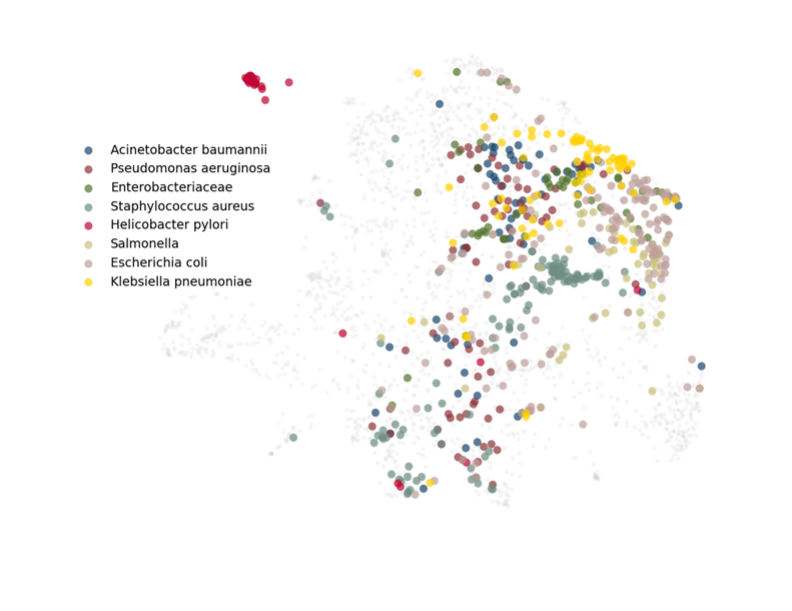
The composite UMAP (Uniform Manifold Approximation and Projection) of paper title embeddings for the tweeted articles. The articles associated with each pathogen are plotted in different colors.

Many factors affect the ability of an organism to garner greater web-based attention than others. We hypothesized that organisms that were positively correlated with URL clicks would be published under a greater range of topics, therefore attracting a greater number of readers. Using a UMAP (Uniform Manifold Approximation and Projection) 2-dimensional vector mapping [20], we created a visual representation of the diversity of paper topics for the selected organisms, whose names appear in more than 30 tweeted papers (Figure 3) and each organism individually (Figure S6 in Multimedia Appendix 1). The papers about *E coli* are broadly distributed in the embedding space as much as those about *P aeruginosa*, but *E coli* is not associated with either an increase or decrease in URL clicks, while *P aeruginosa* is at the level of 0.05 in our regressions. This naive comparison suggests that the extent to which papers about a pathogen are distributed across topics is unrelated to URL clicks. More analysis may be needed to determine whether topic diversity is a factor, and this will be the focus of future studies.

### Do Particular Article Types Receive More URL Clicks?

It is clear that the mention of particular pathogens has a significant effect on the level of user engagement a tweet will receive. However, to assess the impact of the type of paper on the number of URL clicks, we manually categorized the tweets with the highest number of URL clicks based on their general focus (Figure S7 in Multimedia Appendix 1). Almost half of the assessed tweets (48%) focused on the basic science of antibiotic resistance or resistant organisms. When the number of URL clicks was also considered, the tweets with over 30 URL clicks showed a more even distribution between article types, with reviews comprising 31% of all tweets with high URL clicks, as expected [21]. As URL clicks decreased, fundamental science papers became dominant, comprising 34% and 47% of papers with 20-29 URL clicks and 11-19 URL clicks, respectively.

## Discussion

In 2017, the WHO published a list of organisms or bacterial families that pose the greatest risk to human health and for which new treatments are urgently needed. In recent years, these pathogens have been the subject of a significant volume of research. In this study, we investigated and characterized how research papers on these pathogens are consumed and engaged with on Twitter by leveraging the data available via the in-built Twitter analytics toolset on the @AntibioticResis account and data available through the Twitter Academic API. This revealed that certain pathogens are positively associated with URL clicks, while other priority pathogens do not receive the anticipated levels of engagement.

In order to ground our results in the correct context, we characterized both the academic and general interests of each of the WHO priority pathogens and 3 organisms of specific interest. The data presented in Figure 2 show that academic interest in most of the priority pathogens is relatively stable or has experienced minor increases. Two pathogens (*A. baumannii* and *K. pneumoniae*) had a major increase in academic interest during this time. This is perhaps understandable for *A. baumannii* given its position at the top of the WHO priority pathogen list; however, the driver for academic interest in *K. pneumoniae* is less clear. Two priority pathogens (*S. pneumoniae* and *H. influenzae*) lost academic attention in this window, and the driver for this is not clear. This drop in academic attention may need to be addressed with more targeted funding instruments to stimulate research on these pathogens. Within general interest, there is an overall increase in all pathogens. However, it was noted that several organisms showed periodic spikes in interest from the general public on Twitter, with mentions of the organism increasing. Using data gathered from Google Trends and using *Shigella* as an example of one such spike, we showed that the increased interest on Twitter in 2015 was correlated to a spike in searches for the organism on Google at the same time. It is noteworthy that at this time there was a confirmed outbreak of *Shigella* in San Diego, affecting 190 people (Food Safety News, 2015). This provides evidence that real-world events have the capacity to drive interest in particular organisms on social media.

To understand the types of followers of the @AntibioticResis Twitter account, we assessed the number of followers gained by the account each month. We observe a clear spike in new follower numbers in November of each year. We hypothesize that these spikes in follower numbers are due to increased awareness of the account during the annual global education and outreach event “World Antibiotic Awareness Week” (WAAW), which is run by the WHO in November each year. This suggests that not only are internal factors, such as engagement with an account, able to attract new followers but also external factors, such as WAAW, may also encourage this. This agrees with the findings of Dyar et al [9], who determined that institutional events can amplify interest in antibiotic resistance–related content on Twitter; however, this increased interest is short-lived [8].

We then aimed to show the professional background of the followers of the @AntibioticResis account. Our findings indicate a strong follower base rooted in professional medical and academic practice, highlighting how, in busy professional environments, Twitter bots such as this page may offer a fast and accessible gateway to staying abreast of the latest developments in this field. It is expected that students make up a large proportion of the follower base as Twitter users are younger compared to the general US public, according to the Pew Research Center [22]. In recent years, particularly during the COVID-19 pandemic and the age of distance learning, there has been a push to find alternative methods of engaging students in higher education, and Twitter is no exception. Many studies have assessed the potential of Twitter as a method to promote learning among medical students, nursing students, and postgraduate students in general [23-25]. These factors may drive students to use the platform to engage with educational material and stay up-to-date with their scientific areas of interest.

The impact that the inclusion of individual pathogens has on the engagement a tweet receives was examined. Interestingly, even after controlling for the influence of academic and general attention, 3 critical priority pathogens, *A. baumannii*, *P. aeruginosa*, and Enterobacteriaceae, were positively associated with URL clicks. In addition, one pathogen of specific interest, *K. pneumoniae*, was positively associated with URL clicks. Moreover, our data suggest that academic interest influences URL clicks more than general interest, as was expected. Given the increased academic activity described previously for *A. baumannii* and *K. pneumonia*, this is likely reflected in engagement with associated content on Twitter. *P. aeruginosa* has only experienced a marginal increase in academic attention in the last 30 years; however, it represents one of the most widely published organisms in microbiology, and thus this consistent high-volume output has likely garnered a large captive audience responsible for consistently high content engagement levels. One factor that was not accounted for in our study is the influence of high-profile users termed “sirens,” who may be institutions with an active digital outreach program or individual academics who are strong communicators and could act as potent amplifiers of content relevant to their field, therefore artificially driving user engagement. Future work will explore the role of these individuals in driving pathogen academic and general interest.

Further, we identified specific nonpathogen-related stems that have an impact on tweet engagement. These data highlight that the selection of words when creating a title for a research paper can significantly impact the levels of engagement the paper will receive on social media. This is particularly interesting when considering 2 stems that could have the same meaning, such as “antimicrobi” and “agent.” In the case of these 2 stems, the selection of one word over the other will significantly positively or negatively impact the engagements on social media. In some articles, these words may be used interchangeably, and this could have significant consequences for the performance of the paper if posted on social media sites. It should be noted that a low *R*^2^ does not discount the findings of our work. Building a regression model with a high *R*^2^ is required if the model aims to predict a value with low errors. However, the goal of this study is to identify pathogens that significantly affect URL clicks. The statistical significance of covariates is the outcome to be interpreted for this type of study, commonly found in social science [26].

Finally, we assessed how the type of paper included in a tweet may affect web-based engagement. Interestingly, our data show that the percentage of clinically focused papers remains constant across the high, moderate, and low URL click groups, while review articles constitute the largest portion of the high URL click group.

Although the data presented here presents some interesting findings, the authors acknowledge some limitations of this work. Due to the nature of the in-built Twitter Analytics toolset, we only had access to tweet engagement data from December 2018 onward. This means that, relative to the lifespan of the account, the data presented here represent a snapshot of the total engagements. In addition to the limited timeframe discussed in this study, the @AntibioticResis Twitter bot only posts links to publications that appear on PubMed. This means that the account does not post preprint articles. With the recent interest in making preprint articles available on the internet and evidence suggesting that releasing preprint articles can improve the performance of the peer-reviewed article [27-29], this may mean that our analysis is limited or that important interactions are missed. However, while not directly applicable to all fields, the information gleaned from the @AntibioticResis account may act as a guide for academics and clinicians who wish to increase user engagement with their publications and promote antimicrobial stewardship on Twitter.

Overall, this work sheds light on the key interaction kinetics of specific priority pathogens. It also offers a blueprint for how researchers can create a title for their research publication that will maximize the levels of engagement it is likely to achieve on Twitter. Maximizing engagement with research on Twitter can have multiple benefits, including raising the profile of the research and the researchers, increasing citations, and generating outreach and mainstream media opportunities.

## Appendix

Multimedia Appendix 1 Supplementary data.

## Abbreviations

AMR: antimicrobial resistance
API: application programming interface
WAAW: World Antibiotic Awareness Week
WHO: World Health Organization
NB: negative binomial

## Data Availability

The data used in this study are either publicly available on Twitter or can be requested from the corresponding author.
