# Supplementary material for: Understanding the Consumption of Antimicrobial Resistance–Related Content on Social Media: Twitter Analysis
Source: J Med Internet Res. 2023 Jun 12;25:e42363. doi: 10.2196/42363 (PMC10337475; doi:10.2196/42363)
Supplement: Multimedia Appendix 1 [file jmir_v25i1e42363_app1.docx]

**Understanding the consumption of antimicrobial resistance related content on Social Media**

Hyunuk Kim^1^, Chris R Proctor^2^, Dylan Walker^3^ and Ronan R McCarthy^2^

^1^ Department of Management and Entrepreneurship, Martha and Spencer Love School of Business, Elon University, Elon, North Carolina, USA

^2^ Division of Biosciences, Department of Life Sciences, Centre of Inflammation Research and Translational Medicine, College of Health and Life Sciences, Brunel University London, Uxbridge, UB8 3PH, UK.

^3^ The George L. Argyros School of Business and Economics, Chapman University, Orange, California, USA


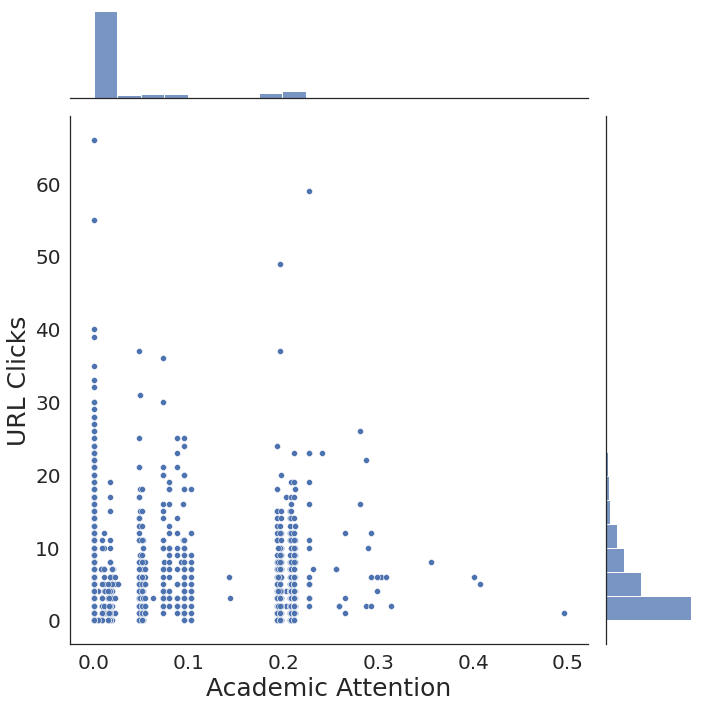


**Figure S1** - The distribution of academic attention across publications.


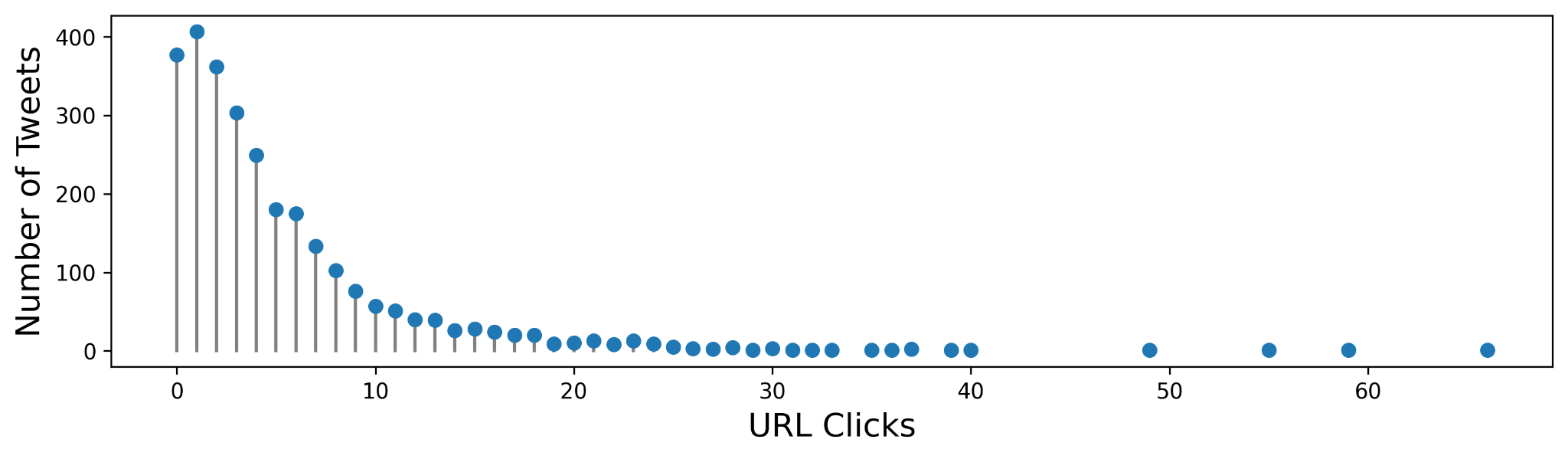


**Figure S2** - The distribution for URL clicks that the tweets by @AntibioticResis received.


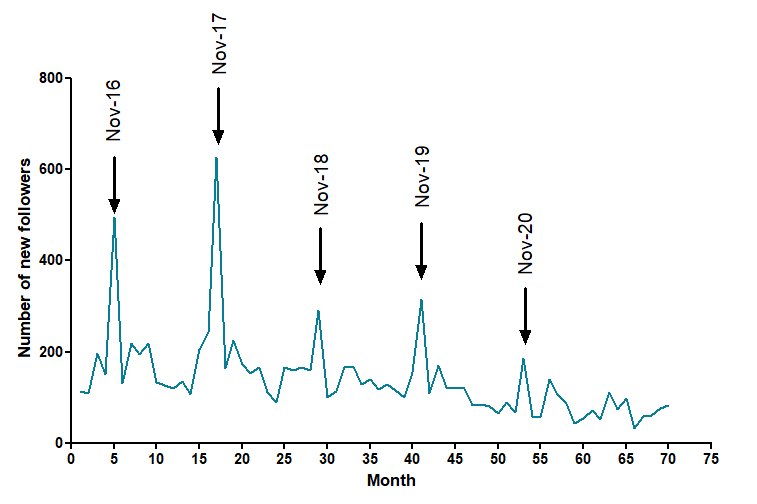


**Figure S3** - Trend in the number of followers of the @AntibioticResis twitter account from July 2016 to April 2022. Noticeable spikes in new follower numbers occur in November of each year. This is likely due to the occurrence of the WHO global campaign “World Antibiotic Awareness Week” which occurs annually in November increasing awareness of the account.


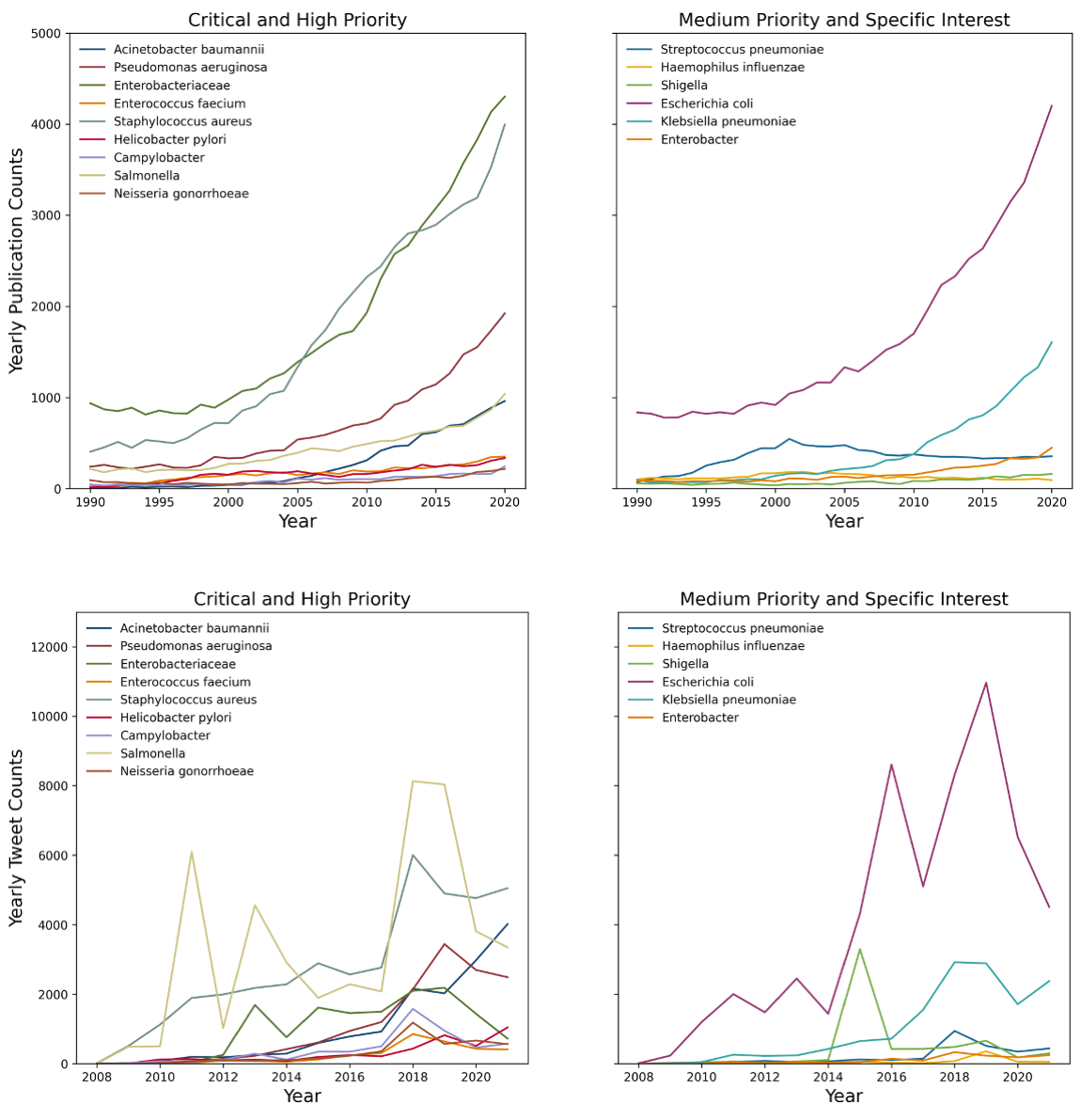


**Figure S4** - (Top) The number of publications made available per year on PubMed regarding the selected pathogens and antimicrobial resistance. (Bottom) The number of tweets per year mentioning one of the selected pathogens.


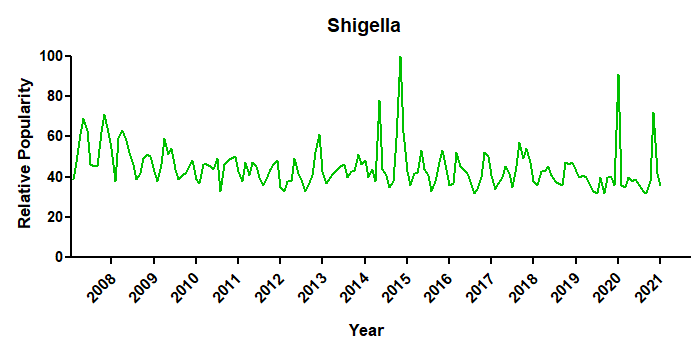


**Figure S5** - Google Trends data showing the relative popularity of the search term “Shigella” over time. A clear peak in searches occurs in 2015 when searches for this term were at their highest within the 2008-2021-time frame.


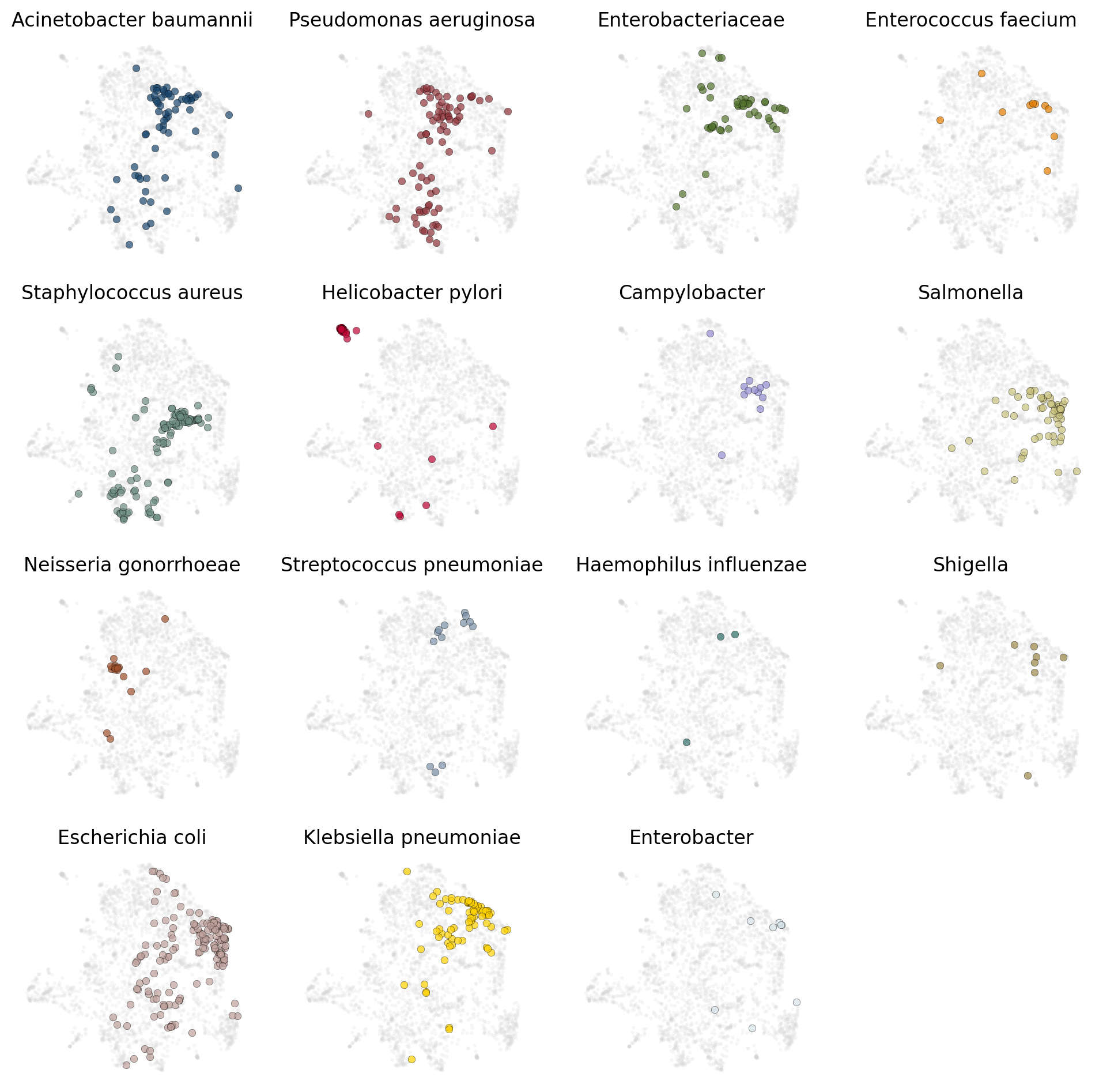


**Figure S6** - Individual UMAP projections of paper title embeddings for the tweeted articles regarding the selected pathogens.

##
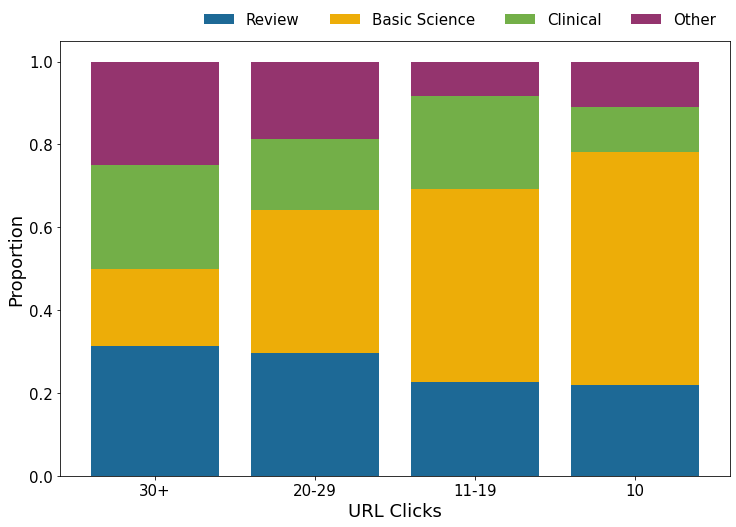


**Figure S7** - The proportion of articles comprising each set of high URL clicks. A relatively even distribution of article types was seen in the highest URL click group while basic science papers became more prevalent with decreasing URL clicks.

**Table S1** - A summary of the terms used to categorize followers as clinically focused or academically focused.

| Health Professionals / Practitioners | clinical |
| --- | --- |
|  | md |
|  | medical |
|  | medicine |
|  | nurse |
|  | pharmacist |
|  | pharmacy |
|  | physician |
| Academic | assistant |
|  | associate |
|  | candidate |
|  | fellow |
|  | institute |
|  | lecturer |
|  | phd |
|  | professor |
|  | reader |
|  | research |
|  | researcher |
|  | scientist |
|  | student |
|  | university |

**Table S2** - A summary of the terms used to categorize followers based on their focus or area of interest.

| Antimicrobial resistance | amr |
| --- | --- |
|  | antibiotic |
|  | antimicrobial |
|  | resistance |
|  | stewardship |
| Infectious Disease | control |
|  | disease |
|  | diseases |
|  | infection |
|  | infectious |
|  | prevention |
| Microbiology | microbiologist |
|  | microbiology |
|  | molecular |
| Public Health | care |
|  | food |
|  | global |
|  | health |
|  | healthcare |
|  | policy |
|  | public |
|  | safety |

**Table S3** - The full regression result for Model 1. Rate ratios can be calculated by exponentiating coefficients.

| Dependent variable: URL clicks  Model: Negative binomial  Method: MLE  Number of observations: 2762 | | | Pseudo R-squared: 0.074  Log-Likelihood: -6908.6  LL-Null: -7456.8  LLR p-value: 3.759e-177 | | | |
| --- | --- | --- | --- | --- | --- | --- |
|  | coef | std err | z | P>\|z\| | [0.025 | 0.975] |
| const | 1.70 | 0.05 | 31.74 | 0.00 | 1.59 | 1.80 |
| acinetobacter baumannii | 0.33 | 0.08 | 3.92 | 0.00 | 0.16 | 0.49 |
| pseudomonas aeruginosa | 0.28 | 0.08 | 3.42 | 0.00 | 0.12 | 0.44 |
| enterobacteriaceae | 0.61 | 0.10 | 6.37 | 0.00 | 0.42 | 0.79 |
| enterococcus faecium | 0.10 | 0.23 | 0.45 | 0.66 | -0.34 | 0.55 |
| staphylococcus aureus | 0.28 | 0.07 | 4.13 | 0.00 | 0.15 | 0.42 |
| helicobacter pylori | -0.22 | 0.15 | -1.46 | 0.15 | -0.51 | 0.08 |
| campylobacter | -0.24 | 0.26 | -0.94 | 0.35 | -0.75 | 0.26 |
| salmonella | 0.07 | 0.11 | 0.60 | 0.55 | -0.15 | 0.29 |
| neisseria gonorrhoeae | -0.31 | 0.21 | -1.47 | 0.14 | -0.71 | 0.10 |
| streptococcus pneumoniae | 0.01 | 0.25 | 0.03 | 0.98 | -0.47 | 0.49 |
| haemophilus influenzae | -1.27 | 0.96 | -1.32 | 0.19 | -3.15 | 0.62 |
| shigella | -0.06 | 0.30 | -0.20 | 0.84 | -0.64 | 0.52 |
| escherichia coli | 0.24 | 0.06 | 3.94 | 0.00 | 0.12 | 0.37 |
| klebsiella pneumoniae | 0.48 | 0.07 | 6.64 | 0.00 | 0.34 | 0.62 |
| enterobacter | 0.43 | 0.24 | 1.79 | 0.07 | -0.04 | 0.90 |
| infect | 0.10 | 0.05 | 1.90 | 0.06 | 0.00 | 0.20 |
| treatment | 0.22 | 0.06 | 3.43 | 0.00 | 0.09 | 0.34 |
| new | 0.30 | 0.09 | 3.26 | 0.00 | 0.12 | 0.48 |
| antibiot | 0.22 | 0.04 | 4.93 | 0.00 | 0.13 | 0.30 |
| mechan | 0.05 | 0.09 | 0.59 | 0.56 | -0.12 | 0.23 |
| resist | 0.12 | 0.04 | 3.11 | 0.00 | 0.05 | 0.20 |
| factor | 0.14 | 0.11 | 1.35 | 0.18 | -0.07 | 0.35 |
| novel | 0.10 | 0.09 | 1.12 | 0.26 | -0.08 | 0.29 |
| agent | -0.31 | 0.13 | -2.40 | 0.02 | -0.57 | -0.06 |
| virul | 0.01 | 0.10 | 0.12 | 0.90 | -0.18 | 0.20 |
| develop | -0.03 | 0.11 | -0.24 | 0.81 | -0.24 | 0.19 |
| clinic | 0.09 | 0.07 | 1.47 | 0.14 | -0.03 | 0.22 |
| associ | 0.14 | 0.08 | 1.67 | 0.09 | -0.02 | 0.30 |
| studi | -0.04 | 0.07 | -0.61 | 0.54 | -0.17 | 0.09 |
| strain | -0.10 | 0.09 | -1.14 | 0.25 | -0.28 | 0.07 |
| gene | 0.02 | 0.06 | 0.27 | 0.79 | -0.10 | 0.14 |
| effect | -0.01 | 0.09 | -0.18 | 0.86 | -0.18 | 0.15 |
| preval | 0.02 | 0.09 | 0.24 | 0.81 | -0.15 | 0.19 |
| bacteri | 0.13 | 0.07 | 1.84 | 0.07 | -0.01 | 0.26 |
| mutat | -0.01 | 0.13 | -0.07 | 0.95 | -0.26 | 0.24 |
| antibacteri | 0.04 | 0.10 | 0.38 | 0.71 | -0.16 | 0.24 |
| detect | 0.19 | 0.08 | 2.39 | 0.02 | 0.04 | 0.35 |
| diseas | 0.13 | 0.11 | 1.22 | 0.22 | -0.08 | 0.34 |
| children | -0.23 | 0.13 | -1.72 | 0.09 | -0.49 | 0.03 |
| sequenc | 0.17 | 0.10 | 1.64 | 0.10 | -0.03 | 0.37 |
| isol | 0.03 | 0.06 | 0.57 | 0.57 | -0.08 | 0.14 |
| suscept | -0.07 | 0.09 | -0.77 | 0.44 | -0.23 | 0.10 |
| inhibitor | 0.01 | 0.10 | 0.06 | 0.95 | -0.19 | 0.20 |
| patient | 0.10 | 0.06 | 1.75 | 0.08 | -0.01 | 0.22 |
| vitro | 0.01 | 0.12 | 0.07 | 0.95 | -0.22 | 0.24 |
| review | -0.01 | 0.08 | -0.11 | 0.92 | -0.17 | 0.15 |
| character | -0.16 | 0.10 | -1.63 | 0.10 | -0.36 | 0.03 |
| china | -0.11 | 0.11 | -0.96 | 0.34 | -0.33 | 0.11 |
| antimicrobi | 0.36 | 0.05 | 7.89 | 0.00 | 0.27 | 0.45 |
| molecular | 0.17 | 0.09 | 2.01 | 0.05 | 0.00 | 0.34 |
| activ | -0.21 | 0.08 | -2.72 | 0.01 | -0.37 | -0.06 |
| hospit | 0.08 | 0.07 | 1.05 | 0.30 | -0.07 | 0.22 |
| evalu | -0.08 | 0.11 | -0.75 | 0.45 | -0.30 | 0.13 |
| caus | 0.03 | 0.10 | 0.29 | 0.77 | -0.17 | 0.23 |
| viru | -0.31 | 0.16 | -2.00 | 0.05 | -0.61 | -0.01 |
| system | 0.06 | 0.10 | 0.59 | 0.56 | -0.14 | 0.25 |
| analysi | -0.16 | 0.09 | -1.77 | 0.08 | -0.34 | 0.02 |
| mycobacterium | -0.26 | 0.13 | -1.96 | 0.05 | -0.52 | 0.00 |
| tuberculosi | 0.03 | 0.11 | 0.28 | 0.78 | -0.18 | 0.24 |
| cell | -0.25 | 0.13 | -1.94 | 0.05 | -0.50 | 0.00 |
| risk | -0.15 | 0.13 | -1.22 | 0.22 | -0.40 | 0.09 |
| tract | 0.06 | 0.17 | 0.37 | 0.71 | -0.27 | 0.39 |
| urinari | 0.35 | 0.18 | 1.98 | 0.05 | 0.00 | 0.69 |
| bacteria | 0.30 | 0.07 | 4.65 | 0.00 | 0.17 | 0.43 |
| emerg | 0.23 | 0.09 | 2.52 | 0.01 | 0.05 | 0.40 |
| potenti | -0.02 | 0.09 | -0.24 | 0.81 | -0.19 | 0.15 |
| care | 0.13 | 0.10 | 1.33 | 0.18 | -0.06 | 0.32 |
| unit | 0.02 | 0.12 | 0.16 | 0.87 | -0.22 | 0.26 |
| human | 0.14 | 0.09 | 1.55 | 0.12 | -0.04 | 0.32 |
| use | 0.24 | 0.08 | 2.80 | 0.01 | 0.07 | 0.40 |
| drug | -0.13 | 0.08 | -1.64 | 0.10 | -0.29 | 0.03 |
| impact | 0.02 | 0.10 | 0.26 | 0.80 | -0.16 | 0.21 |
| therapi | 0.01 | 0.09 | 0.12 | 0.91 | -0.16 | 0.18 |
| multidrug-resist | 0.17 | 0.07 | 2.36 | 0.02 | 0.03 | 0.30 |
| genom | -0.10 | 0.10 | -1.02 | 0.31 | -0.30 | 0.09 |
| pathogen | 0.04 | 0.08 | 0.51 | 0.61 | -0.12 | 0.20 |
| biofilm | -0.12 | 0.11 | -1.09 | 0.28 | -0.33 | 0.09 |
| type | 0.20 | 0.11 | 1.91 | 0.06 | -0.01 | 0.42 |
| profil | -0.03 | 0.12 | -0.25 | 0.80 | -0.27 | 0.21 |
| gram-neg | 0.38 | 0.10 | 4.02 | 0.00 | 0.20 | 0.57 |
| n_retweets | 0.21 | 0.01 | 25.14 | 0.00 | 0.20 | 0.23 |
| n_words | -0.08 | 0.01 | -13.24 | 0.00 | -0.09 | -0.07 |
| alpha | 2.51 | 0.11 | 23.00 | 0.00 | 2.30 | 2.72 |

**Table S4** - The full regression result for Model 2. Rate ratios can be calculated by exponentiating coefficients.

| Dependent variable: URL clicks  Model: Negative binomial  Method: MLE  Number of observations: 2762 | | | Pseudo R-squared: 0.074  Log-Likelihood: -6907.6  LL-Null: -7456.8  LLR p-value: 5.705e-177 | | | |
| --- | --- | --- | --- | --- | --- | --- |
|  | coef | std err | z | P>\|z\| | [0.025 | 0.975] |
| const | 1.69 | 0.05 | 31.58 | 0.00 | 1.59 | 1.80 |
| acinetobacter baumannii | 0.71 | 0.29 | 2.46 | 0.01 | 0.14 | 1.28 |
| pseudomonas aeruginosa | 1.04 | 0.56 | 1.87 | 0.06 | -0.05 | 2.13 |
| enterobacteriaceae | 2.25 | 1.19 | 1.89 | 0.06 | -0.08 | 4.59 |
| enterococcus faecium | 0.24 | 0.25 | 0.97 | 0.33 | -0.25 | 0.73 |
| staphylococcus aureus | 1.80 | 1.10 | 1.63 | 0.10 | -0.36 | 3.95 |
| helicobacter pylori | -0.09 | 0.17 | -0.51 | 0.61 | -0.43 | 0.25 |
| campylobacter | -0.16 | 0.26 | -0.62 | 0.53 | -0.68 | 0.35 |
| salmonella | 0.47 | 0.31 | 1.51 | 0.13 | -0.14 | 1.07 |
| neisseria gonorrhoeae | -0.23 | 0.22 | -1.07 | 0.28 | -0.65 | 0.19 |
| streptococcus pneumoniae | 0.15 | 0.27 | 0.55 | 0.58 | -0.37 | 0.67 |
| haemophilus influenzae | -1.22 | 0.96 | -1.27 | 0.20 | -3.11 | 0.66 |
| shigella | 0.01 | 0.30 | 0.03 | 0.97 | -0.58 | 0.60 |
| escherichia coli | 1.86 | 1.18 | 1.59 | 0.11 | -0.44 | 4.17 |
| klebsiella pneumoniae | 1.10 | 0.46 | 2.42 | 0.02 | 0.21 | 1.99 |
| enterobacter | 0.58 | 0.26 | 2.20 | 0.03 | 0.06 | 1.10 |
| infect | 0.10 | 0.05 | 1.86 | 0.06 | -0.01 | 0.20 |
| treatment | 0.22 | 0.06 | 3.45 | 0.00 | 0.09 | 0.34 |
| new | 0.30 | 0.09 | 3.23 | 0.00 | 0.12 | 0.48 |
| antibiot | 0.22 | 0.04 | 4.93 | 0.00 | 0.13 | 0.30 |
| mechan | 0.06 | 0.09 | 0.64 | 0.52 | -0.12 | 0.23 |
| resist | 0.12 | 0.04 | 3.08 | 0.00 | 0.04 | 0.20 |
| factor | 0.15 | 0.11 | 1.36 | 0.17 | -0.06 | 0.35 |
| novel | 0.10 | 0.09 | 1.13 | 0.26 | -0.08 | 0.29 |
| agent | -0.31 | 0.13 | -2.35 | 0.02 | -0.56 | -0.05 |
| virul | 0.00 | 0.10 | 0.02 | 0.98 | -0.19 | 0.20 |
| develop | -0.02 | 0.11 | -0.23 | 0.82 | -0.24 | 0.19 |
| clinic | 0.09 | 0.06 | 1.45 | 0.15 | -0.03 | 0.22 |
| associ | 0.14 | 0.08 | 1.71 | 0.09 | -0.02 | 0.30 |
| studi | -0.04 | 0.07 | -0.58 | 0.56 | -0.17 | 0.09 |
| strain | -0.09 | 0.09 | -0.97 | 0.33 | -0.27 | 0.09 |
| gene | 0.02 | 0.06 | 0.30 | 0.76 | -0.10 | 0.14 |
| effect | -0.02 | 0.09 | -0.18 | 0.86 | -0.18 | 0.15 |
| preval | 0.02 | 0.09 | 0.20 | 0.84 | -0.15 | 0.18 |
| bacteri | 0.12 | 0.07 | 1.83 | 0.07 | -0.01 | 0.26 |
| mutat | -0.01 | 0.13 | -0.08 | 0.94 | -0.26 | 0.24 |
| antibacteri | 0.04 | 0.10 | 0.38 | 0.71 | -0.16 | 0.24 |
| detect | 0.19 | 0.08 | 2.37 | 0.02 | 0.03 | 0.35 |
| diseas | 0.12 | 0.11 | 1.15 | 0.25 | -0.09 | 0.33 |
| children | -0.25 | 0.14 | -1.82 | 0.07 | -0.51 | 0.02 |
| sequenc | 0.17 | 0.10 | 1.59 | 0.11 | -0.04 | 0.37 |
| isol | 0.03 | 0.06 | 0.56 | 0.58 | -0.08 | 0.14 |
| suscept | -0.06 | 0.09 | -0.75 | 0.45 | -0.23 | 0.10 |
| inhibitor | 0.01 | 0.10 | 0.11 | 0.91 | -0.18 | 0.21 |
| patient | 0.11 | 0.06 | 1.80 | 0.07 | -0.01 | 0.22 |
| vitro | 0.01 | 0.12 | 0.08 | 0.94 | -0.22 | 0.24 |
| review | -0.02 | 0.08 | -0.20 | 0.84 | -0.18 | 0.14 |
| character | -0.17 | 0.10 | -1.69 | 0.09 | -0.36 | 0.03 |
| china | -0.11 | 0.11 | -0.99 | 0.32 | -0.34 | 0.11 |
| antimicrobi | 0.36 | 0.05 | 7.89 | 0.00 | 0.27 | 0.45 |
| molecular | 0.17 | 0.09 | 2.03 | 0.04 | 0.01 | 0.34 |
| activ | -0.21 | 0.08 | -2.71 | 0.01 | -0.37 | -0.06 |
| hospit | 0.07 | 0.07 | 1.00 | 0.32 | -0.07 | 0.22 |
| evalu | -0.08 | 0.11 | -0.73 | 0.47 | -0.30 | 0.14 |
| caus | 0.04 | 0.10 | 0.38 | 0.70 | -0.16 | 0.24 |
| viru | -0.31 | 0.16 | -2.00 | 0.05 | -0.61 | -0.01 |
| system | 0.06 | 0.10 | 0.59 | 0.56 | -0.14 | 0.25 |
| analysi | -0.16 | 0.09 | -1.82 | 0.07 | -0.34 | 0.01 |
| mycobacterium | -0.26 | 0.13 | -1.95 | 0.05 | -0.52 | 0.00 |
| tuberculosi | 0.03 | 0.11 | 0.27 | 0.79 | -0.18 | 0.24 |
| cell | -0.25 | 0.13 | -1.94 | 0.05 | -0.50 | 0.00 |
| risk | -0.15 | 0.13 | -1.21 | 0.23 | -0.40 | 0.10 |
| tract | 0.06 | 0.17 | 0.35 | 0.73 | -0.27 | 0.39 |
| urinari | 0.34 | 0.18 | 1.95 | 0.05 | 0.00 | 0.69 |
| bacteria | 0.30 | 0.07 | 4.63 | 0.00 | 0.17 | 0.43 |
| emerg | 0.22 | 0.09 | 2.49 | 0.01 | 0.05 | 0.40 |
| potenti | -0.02 | 0.09 | -0.26 | 0.80 | -0.19 | 0.15 |
| care | 0.13 | 0.10 | 1.31 | 0.19 | -0.06 | 0.32 |
| unit | 0.02 | 0.12 | 0.14 | 0.89 | -0.23 | 0.26 |
| human | 0.14 | 0.09 | 1.55 | 0.12 | -0.04 | 0.32 |
| use | 0.24 | 0.08 | 2.81 | 0.01 | 0.07 | 0.40 |
| drug | -0.13 | 0.08 | -1.65 | 0.10 | -0.29 | 0.03 |
| impact | 0.02 | 0.10 | 0.26 | 0.79 | -0.16 | 0.21 |
| therapi | 0.01 | 0.09 | 0.11 | 0.92 | -0.16 | 0.18 |
| multidrug-resist | 0.16 | 0.07 | 2.35 | 0.02 | 0.03 | 0.30 |
| genom | -0.10 | 0.10 | -0.96 | 0.34 | -0.29 | 0.10 |
| pathogen | 0.04 | 0.08 | 0.51 | 0.61 | -0.12 | 0.20 |
| biofilm | -0.12 | 0.11 | -1.13 | 0.26 | -0.33 | 0.09 |
| type | 0.20 | 0.11 | 1.90 | 0.06 | -0.01 | 0.41 |
| profil | -0.04 | 0.12 | -0.29 | 0.77 | -0.28 | 0.21 |
| gram-neg | 0.38 | 0.10 | 4.01 | 0.00 | 0.20 | 0.57 |
| n_retweets | 0.21 | 0.01 | 25.09 | 0.00 | 0.20 | 0.23 |
| n_words | -0.08 | 0.01 | -13.20 | 0.00 | -0.09 | -0.07 |
| academic_attention | -7.76 | 5.62 | -1.38 | 0.17 | -18.78 | 3.25 |
| alpha | 2.51 | 0.11 | 22.98 | 0.00 | 2.29 | 2.72 |

**Table S5** - The full regression result for Model 3. Rate ratios can be calculated by exponentiating coefficients.

| Dependent variable: URL clicks  Model: Negative binomial  Method: MLE  Number of observations: 2762 | | | Pseudo R-squared: 0.072  Log-Likelihood: -6921.4  LL-Null: -7456.8  LLR p-value: 2.004e-171 | | | |
| --- | --- | --- | --- | --- | --- | --- |
|  | coef | std err | z | P>\|z\| | [0.025 | 0.975] |
| const | 1.62 | 0.05 | 30.19 | 0.00 | 1.52 | 1.73 |
| acinetobacter baumannii | 0.52 | 0.15 | 3.49 | 0.00 | 0.23 | 0.82 |
| pseudomonas aeruginosa | 0.39 | 0.14 | 2.75 | 0.01 | 0.11 | 0.66 |
| enterobacteriaceae | 0.70 | 0.14 | 5.16 | 0.00 | 0.43 | 0.97 |
| enterococcus faecium | 0.10 | 0.29 | 0.35 | 0.73 | -0.47 | 0.67 |
| staphylococcus aureus | 0.41 | 0.19 | 2.20 | 0.03 | 0.04 | 0.78 |
| helicobacter pylori | -0.09 | 0.15 | -0.58 | 0.56 | -0.38 | 0.21 |
| campylobacter | -0.37 | 0.28 | -1.29 | 0.20 | -0.92 | 0.19 |
| salmonella | 0.23 | 0.22 | 1.05 | 0.29 | -0.20 | 0.65 |
| neisseria gonorrhoeae | -0.31 | 0.23 | -1.35 | 0.18 | -0.75 | 0.14 |
| streptococcus pneumoniae | -0.05 | 0.26 | -0.21 | 0.84 | -0.57 | 0.46 |
| haemophilus influenzae | -0.82 | 0.65 | -1.25 | 0.21 | -2.10 | 0.46 |
| shigella | 0.00 | 0.31 | 0.00 | 1.00 | -0.61 | 0.61 |
| escherichia coli | 0.41 | 0.26 | 1.55 | 0.12 | -0.11 | 0.93 |
| klebsiella pneumoniae | 0.53 | 0.13 | 4.19 | 0.00 | 0.28 | 0.78 |
| enterobacter | 0.33 | 0.30 | 1.13 | 0.26 | -0.25 | 0.91 |
| infect | 0.11 | 0.06 | 1.92 | 0.06 | 0.00 | 0.22 |
| treatment | 0.14 | 0.08 | 1.81 | 0.07 | -0.01 | 0.28 |
| new | 0.37 | 0.11 | 3.44 | 0.00 | 0.16 | 0.58 |
| antibiot | 0.26 | 0.05 | 5.18 | 0.00 | 0.16 | 0.35 |
| mechan | -0.03 | 0.11 | -0.24 | 0.81 | -0.24 | 0.19 |
| resist | 0.12 | 0.04 | 2.90 | 0.00 | 0.04 | 0.21 |
| factor | 0.11 | 0.13 | 0.84 | 0.40 | -0.14 | 0.36 |
| novel | 0.09 | 0.10 | 0.84 | 0.40 | -0.12 | 0.29 |
| agent | -0.48 | 0.14 | -3.44 | 0.00 | -0.76 | -0.21 |
| virul | 0.04 | 0.12 | 0.36 | 0.72 | -0.18 | 0.27 |
| develop | -0.01 | 0.12 | -0.08 | 0.94 | -0.25 | 0.23 |
| clinic | 0.15 | 0.07 | 2.02 | 0.04 | 0.00 | 0.29 |
| associ | 0.06 | 0.10 | 0.67 | 0.50 | -0.12 | 0.25 |
| studi | -0.06 | 0.07 | -0.87 | 0.38 | -0.21 | 0.08 |
| strain | -0.13 | 0.10 | -1.22 | 0.22 | -0.33 | 0.08 |
| gene | -0.02 | 0.07 | -0.27 | 0.79 | -0.15 | 0.12 |
| effect | -0.01 | 0.09 | -0.08 | 0.94 | -0.19 | 0.17 |
| preval | 0.04 | 0.10 | 0.37 | 0.71 | -0.15 | 0.22 |
| bacteri | 0.12 | 0.08 | 1.58 | 0.11 | -0.03 | 0.28 |
| mutat | -0.08 | 0.13 | -0.61 | 0.54 | -0.34 | 0.18 |
| antibacteri | 0.07 | 0.11 | 0.65 | 0.52 | -0.15 | 0.30 |
| detect | 0.19 | 0.10 | 1.95 | 0.05 | 0.00 | 0.37 |
| diseas | 0.28 | 0.12 | 2.27 | 0.02 | 0.04 | 0.52 |
| children | -0.20 | 0.13 | -1.50 | 0.13 | -0.46 | 0.06 |
| sequenc | 0.17 | 0.12 | 1.42 | 0.15 | -0.06 | 0.40 |
| isol | -0.05 | 0.07 | -0.84 | 0.40 | -0.18 | 0.07 |
| suscept | 0.01 | 0.09 | 0.11 | 0.92 | -0.17 | 0.19 |
| inhibitor | 0.04 | 0.11 | 0.39 | 0.70 | -0.17 | 0.25 |
| patient | 0.09 | 0.07 | 1.33 | 0.19 | -0.04 | 0.22 |
| vitro | -0.05 | 0.13 | -0.42 | 0.67 | -0.30 | 0.20 |
| review | 0.04 | 0.10 | 0.45 | 0.65 | -0.15 | 0.23 |
| character | -0.24 | 0.11 | -2.21 | 0.03 | -0.45 | -0.03 |
| china | -0.17 | 0.12 | -1.38 | 0.17 | -0.41 | 0.07 |
| antimicrobi | 0.37 | 0.05 | 6.84 | 0.00 | 0.26 | 0.47 |
| molecular | 0.20 | 0.10 | 2.12 | 0.03 | 0.02 | 0.39 |
| activ | -0.28 | 0.09 | -3.27 | 0.00 | -0.45 | -0.11 |
| hospit | 0.21 | 0.09 | 2.47 | 0.01 | 0.04 | 0.38 |
| evalu | -0.11 | 0.12 | -0.95 | 0.34 | -0.34 | 0.12 |
| caus | 0.18 | 0.12 | 1.48 | 0.14 | -0.06 | 0.42 |
| viru | -0.33 | 0.15 | -2.22 | 0.03 | -0.63 | -0.04 |
| system | -0.01 | 0.11 | -0.08 | 0.94 | -0.23 | 0.21 |
| analysi | -0.19 | 0.09 | -2.06 | 0.04 | -0.38 | -0.01 |
| mycobacterium | -0.23 | 0.14 | -1.63 | 0.10 | -0.51 | 0.05 |
| tuberculosi | -0.06 | 0.12 | -0.54 | 0.59 | -0.30 | 0.17 |
| cell | -0.36 | 0.13 | -2.82 | 0.01 | -0.60 | -0.11 |
| risk | -0.07 | 0.14 | -0.47 | 0.64 | -0.35 | 0.21 |
| tract | 0.18 | 0.18 | 0.97 | 0.33 | -0.18 | 0.53 |
| urinari | 0.29 | 0.20 | 1.47 | 0.14 | -0.10 | 0.67 |
| bacteria | 0.32 | 0.08 | 4.33 | 0.00 | 0.18 | 0.47 |
| emerg | 0.34 | 0.12 | 2.82 | 0.01 | 0.10 | 0.57 |
| potenti | -0.07 | 0.10 | -0.71 | 0.48 | -0.27 | 0.12 |
| care | 0.06 | 0.11 | 0.53 | 0.60 | -0.16 | 0.28 |
| unit | -0.05 | 0.14 | -0.34 | 0.73 | -0.33 | 0.23 |
| human | 0.17 | 0.10 | 1.60 | 0.11 | -0.04 | 0.37 |
| use | 0.23 | 0.10 | 2.29 | 0.02 | 0.03 | 0.42 |
| drug | -0.15 | 0.08 | -1.79 | 0.07 | -0.31 | 0.01 |
| impact | 0.01 | 0.10 | 0.10 | 0.92 | -0.19 | 0.22 |
| therapi | -0.01 | 0.09 | -0.14 | 0.89 | -0.20 | 0.17 |
| multidrug-resist | 0.11 | 0.08 | 1.37 | 0.17 | -0.05 | 0.28 |
| genom | -0.03 | 0.11 | -0.31 | 0.76 | -0.24 | 0.18 |
| pathogen | 0.13 | 0.09 | 1.39 | 0.17 | -0.05 | 0.31 |
| biofilm | -0.17 | 0.12 | -1.38 | 0.17 | -0.41 | 0.07 |
| type | 0.16 | 0.13 | 1.29 | 0.20 | -0.08 | 0.41 |
| profil | 0.03 | 0.13 | 0.20 | 0.85 | -0.23 | 0.29 |
| gram-neg | 0.36 | 0.12 | 3.08 | 0.00 | 0.13 | 0.59 |
| n_retweets | 0.24 | 0.01 | 20.30 | 0.00 | 0.22 | 0.27 |
| n_words | -0.08 | 0.01 | -13.76 | 0.00 | -0.09 | -0.07 |
| general_attention | -0.51 | 1.08 | -0.48 | 0.64 | -2.63 | 1.61 |
| alpha | 0.52 | 0.02 | 23.86 | 0.00 | 0.47 | 0.56 |
